# Post-COVID fatigue disproportionately affects women: evidence from the DEFEAT Corona cohort

**DOI:** 10.3389/fpubh.2026.1755106

**Published:** 2026-02-16

**Authors:** Mareike Meier-Maiwald, Tim Riester, Andrea Stölting, Frank Klawonn, Theresa Thölking, Karolin Beinhauer, Viktoria Lampe, Lena-Marie Theil, Dominik Schröder, Georg MN Behrens, Imke Von Wasielewski, Frank Müller, Sandra Steffens, Alexandra Dopfer-Jablonka, Christine Happle, Marie Mikuteit

**Affiliations:** 1 Department of Pediatric Pneumology, Allergology, and Neonatology, Hannover Medical School, Hannover, Germany; 2 Department of Rheumatology and Immunology, Hannover Medical School, Hannover, Germany; 3 Department of General Practice, University Medical Center Göttingen, Göttingen, Germany; 4 Department of Computer Science, Ostfalia University of Applied Sciences, Wolfenbüttel, Germany; 5 Department of Dermatology and Allergy, Hannover Medical School, Hannover, Germany; 6 Department of Medical Education, Hannover Medical School, Hannover, Germany; 7 Department of Family Medicine, Michigan State University, Grand Rapids, MI, United States; 8 German Center for Lung Research, Biomedical Research in Endstage and Obstructive Lung Disease Hannover, Hannover, Germany

**Keywords:** fatigue, gender, long COVID, post COVID syndrome, quality of life, sex, women’s health

## Abstract

**Background:**

Post COVID syndrome (PCS) affects approximately 6–10% of COVID-19 survivors, with fatigue being one of the most prevalent and debilitating symptoms. While emerging evidence suggests sex and gender-based differences in PCS manifestation, the specific impact on fatigue-related symptoms remains poorly understood, particularly regarding women’s experiences.

**Methods:**

We analyzed data from 2,549 participants with PCS (80.6% female, 19.2% male, 0.2% non-binary) from DEFEAT, an online platform surveying people with and without PCS. Participants with confirmed SARS-CoV-2 infection were included if they reported symptoms typical for PCS persisting >4 weeks post-infection. Fatigue-related symptoms including fatigue, brain fog, sleep disturbances, and associated quality of life measures were assessed using validated instruments.

**Results:**

Female participants reported significantly higher rates of fatigue-related symptoms compared to males: fatigue was more prevalent in females (53.5% vs. 46.3%, *p* < 0.001), as were brain fog (54.9% vs. 44.7%, *p* < 0.001), and sleep disturbances (54.8% vs. 45.3%, *p* < 0.001). Female participants also reported significantly higher fatigue severity scores and poorer health-related quality of life (mean EQ-5D score 0.66 (SD 0.23) vs. 0.71 (SD 0.23) in males, *p* < 0.001). Around two thirds of menstruating PCS patients reported menstrual cycle associated worsening of fatigue symptoms.

**Conclusion:**

Our findings demonstrate significant gender-based differences in fatigue-related symptoms in PCS, with women experiencing both higher prevalence and severity, and menstrual cycle associated symptom worsening. These results highlight the importance of sex and gender-specific approaches to understanding and managing PCS-related fatigue, with implications for clinical care and future research directions.

## Background

Post COVID syndrome (PCS) represents a significant global health challenge affecting an estimated 6–10% of COVID-19 survivors (1, 2). This condition is characterized by persistent or new symptoms that continue beyond 4 weeks following acute SARS-CoV-2 infection and are not attributable to another disease, with fatigue being among the most commonly reported and debilitating manifestations (3). Although the molecular mechanisms underlying PCS are not fully understood yet, autoimmunity has been shown to be a hallmark characteristic of this disease (4).

The prevalence and impact of PCS vary considerably across different populations, with emerging evidence suggesting important sex-based differences in symptom presentation and severity (5). Understanding such sex and gender specific differences in PCS is crucial for developing targeted therapeutic approaches and ensuring equitable healthcare delivery.

Recent neuroimaging research identified brain network abnormalities associated with mental fatigue that involve cortical–subcortical networks within frontal, limbic, basal ganglia and parietal structures (6). While acute COVID-19 showed male predominance in hospitalization, severe outcomes and mortality (7, 8), PCS appears to disproportionately affect women, and merging evidence suggests that female predominance in PCS may be linked to sex-specific autoimmune dysregulation, including higher autoantibody levels, enhanced B-cell reactivity, and skewed inflammatory responses in women with persistent symptoms (9). Previous studies demonstrated that female patients are significantly more likely to develop PCS overall, with an up to 50% increased risk compared to males (10, 11). However, the specific manifestation of fatigue-related symptoms and their impact on quality of life in women with PCS remains inadequately characterized.

Fatigue in PCS encompasses not only physical exhaustion, but hallmark symptoms also include cognitive fatigue, brain fog, sleep disturbances, and post-exertional malaise (12, 13). These symptoms can severely impact daily functioning, work productivity, and overall quality of life (14).

The present study aimed to examine gender-based differences in fatigue-related symptoms among individuals with PCS, focusing specifically on the frequency, severity, and quality of life impact of these symptoms, and a possible relation to menstrual cycle phases. By analyzing data from a large German cohort, we sought to provide evidence-based insights that can inform clinical practice and guide future research directions in women’s health and PCS management.

## Methods

### Study design and population

This cross-sectional analysis utilized data from the DEFEAT Corona (Defense Against COVID-19) study, a longitudinal observational study investigating long-term consequences of COVID-19. The study protocol was described in detail elsewhere (15) and approved by the ethics committees of Hannover Medical School (Nr. 9948_BO_K_2021) and University Medical Center Göttingen (29/3/21). The study was registered in the German Clinical Trial Registry (DRKS00026007).

### Participants and data collection

**Figure 1:**
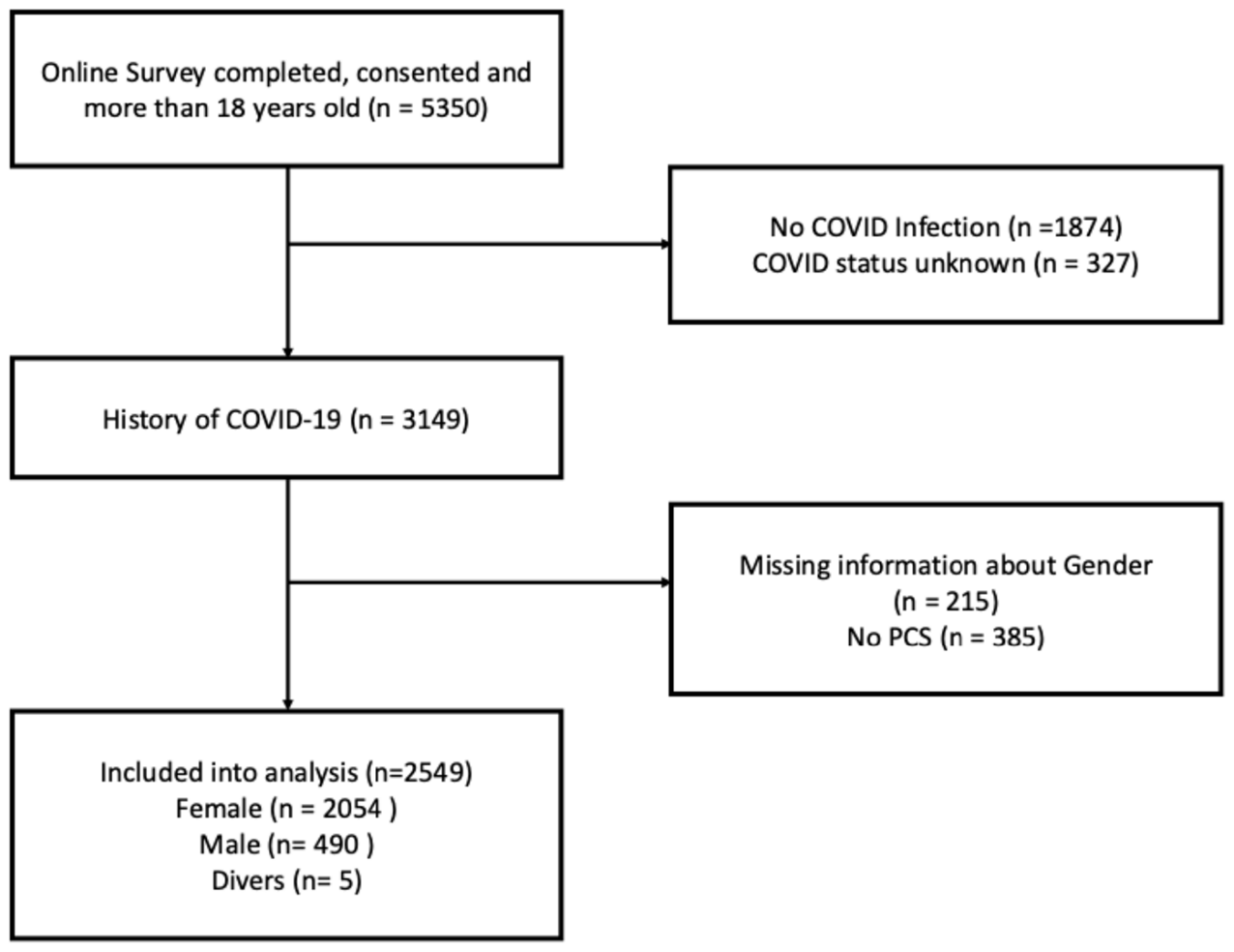
Study flow chart for baseline online survey data.

Participants were recruited via outpatient clinics, cooperation partners, local public health authorities, primary care clinics, support groups, and ministries. Inclusion criteria were: (1) age ≥18 years, (2) written online consent, and (3) confirmed SARS-CoV-2 infection diagnosed by PCR, antigen, or antibody testing and (4) self-reported PCS, defined as persistence or onset of new PCS typical symptoms exceeding 4 weeks after acute SARS-CoV-2 infection and could not be explained otherwise (See Figure 1). Data were collected through an online survey platform (SoSciSurvey) between September 2021 and February 2023, and an additional online survey on menstrual cycle dependent fatigue symptoms was conducted between March and April 2025. Information included demographics, SARS-CoV-2 infection details, ongoing symptoms, vaccination status, and health-related quality of life measures. Of note, these parameters we were including were self-reported. We assessed gender instead of sex which is non-aligning in an estimated 0.4–1.6% of persons (16, 17).

### Assessment of fatigue-related symptoms and quality of life

Fatigue-related symptoms were assessed through self-reported questionnaires focusing on severity and frequency of general fatigue, cognitive symptoms (“brain fog”), and sleep disturbances assessed by a Likert scale from 0 to 10 (0 = no symptoms; 10 = maximum symptoms). A symptom was counted as mentioned if the severity was higher or equal to the median strength of the sample. Symptom severity was calculated in the total PCS sample. Since there is no standardized data on PCS symptom severity thresholds, we used the median for binarization, which is an established concept in biomedical research (18). Health-related quality of life (HRQoL) was measured using the EQ-5D-3L questionnaire, assessing mobility, self-care, everyday activities, pain/discomfort, and anxiety/depression (19). We used the Index scores with a scale from 0 to 1, with 1 representing best health possible, standardized for the German population.

### Statistical analyses

Participant characteristics were presented as median (with interquartile range 25th to 75th percentile, IQR) or mean (with standard deviation, SD) for continuous variables and number (percentage) for categorical variables. Because of the small sample size of the non-binary group, comparisons were only conducted between females and males. Chi-square tests were used for categorical variables, and Wilcoxon-Mann–Whitney tests or t-tests for continuous variables when comparing groups. Linear regression models were used to examine associations between gender (female vs. male) and fatigue severity modeled as a continuous outcome (0–10 Likert scale), adjusting for age (years), vaccination status at the time of infection (yes/no), hospitalization, and time since infection (weeks). A symptom was regarded as definitive per participant if the scaling on the Likert scale was higher or equal to the median severity of the sample. Severity was calculated across all responses. *p*-values were corrected with the Bonferroni-holm method for symptom comparisons. *p*-values <0.05 were considered significant after adjustment. All analyses and figures were conducted using R (version 4.1.2), including the packages tidyverse (version 2.0.0), mosaic (version 1.9.1), epitools (version 0.5–10.1), psych (version 2.4.6.26r), pettyR (version 2.2–3), ggpubr (version 0.6.0), patchwork (version 1.2.0), gtsummary (version 2.4.0).

## Results

### Description of the cohort

A total of 2,549 participants were included into the analysis, of whom 2,054 (80.6%) reported to be female, 490 (19.2%) male, 5 (0.2%) non-binary. Demographics and symptoms for respondents identifying as non-binary are shown in each column, statistics in the far-right column represent comparisons between male and female persons. The median age of all respondents was 43 years (IQR 33–52), and male respondents were slightly older than female. Most participants (61.8%) reported high educational level (college preparatory qualification), with a larger proportion of high educational level in women compared to men. The median time between infection and survey completion was 34.4 weeks (12.1–53.2; Table 1).

**Table 1:** Demographic information by gender.

Parameters	Overall	Female	Male	Non-binary	*p*-value
*n*	2,549	2054	490	5	
Age [median (IQR)]	43 [33–52]	43 [33–52]	45 [34–54]	32 [28–39]	0.009^1^
Not answered, *n* (%)	2 (0.07)	2 (0.09.)	0 (0.0)	0 (0.0)	
School education in years					<0.001^2^
Up to 9 years, *n* (%)	141 (5.6)	86 (4.3)	53 (10.8)	1 (10.0)	
10 years, *n* (%)	830 (32.7)	683 (33.4)	146 (29.8)	1 (20.0)	
A-levels (12 years), *n* (%)	1,571 (61.8)	1,277 (62.4)	291 (59.4)	3 (60.0)	
Not answered, *n* (%)	7 (0.3)	7 (0.30)	0 (0.0)	0 (0.0)	
Time since infection in weeks [median (IQR)]	34.4 [12.1–53.2]	32.8 [10.9–52.1]	34.51 [11.3–53.4]	36.07 [24.6–51.5]	0.149^1^
Not answered, *n* (%)	46 (1.8)	41 (1.78)	17 (2.71)	1 (16.7)	
Hospitalization					
Regular ward, *n* (%)	168 (6.6)	120 (5.8)	48 (9.8)	0 (0.0)	0.002^2^
Intensive care unit, *n* (%)	50 (2.0)	23 (1.1)	27 (5.5)	0 (0.0)	<0.001^2^
Comorbidities (multiple answers)					
Cardiovascular, *n* (%)	455 (17.9)	326 (15.9)	128 (26.1)	1 (20.0)	<0.001^2^
Metabolic, *n* (%)	672 (26.4)	580 (28.2)	91 (18.6)	1 (20.0)	<0.001^2^
Pneumological, *n* (%)	326 (12.8)	273 (13.3)	52 (10.6)	1 (20.0)	0.128^2^
Inflammatory and autoimmune, *n* (%)	339 (13.3)	298 (14.5)	41 (8.4)	0 (0.0)	<0.001^2^
Neuropsychiatric, *n* (%)	517 (20.3)	456 (22.2)	59 (12.0)	2 (40.0)	<0.001^2^
Cancer, *n* (%)	123 (4.8)	105 (5.1)	18 (3.7)	0 (0.0)	0.224^2^
Infection period, *n* (%)					0.027^2^
Until 06/21	1,271 (50.8)	1,009 (50.0)	259 (54.1)	3 (60.0)	
07/2021–12/2021	377 (15.1)	295 (14.6)	81 (16.9)	1 (20.0)	
After 01/2022	854 (34.1)	714 (35.4)	139 (29.0)	1 (20.0)	
Not answered, *n* (%)	47 (1.8)	36 (1.8)	13 (2.7)	0 (0.0)	
Fully vaccinated^*^ against SARS-CoV-2 overall, *n* (%)	1,874 (73.5)	1,519 (74.0)	352 (71.8)	3 (60.0)	0.369^2^
Fully Vaccinated against SARS-CoV-2 prior to infection *n* (%)	1,013 (52.0)	832 (52.9)	180 (48.5)	1 (25.0)	0.148^2^
Not answered, *n* (%)	600 (23.5)	480 (23.4)	119 (24.3)	1 (20.0)	

^1^Wilcoxon and ^2^Chi square test between female and male. *Fully vaccinated was defined as at least two vaccines at time of survey; IQR, Interquartile range.

### Gender-specific PCS and fatigue-related symptoms

Among patients with PCS, female participants demonstrated significantly higher rates of fatigue-related symptoms compared to male participants: 53.5% female vs. 46.3% male persons with PCS reported symptom severity above the sample median (*p* = 0.005, Table 2). Similarly, brain fog was reported more frequently by females (54.8% compared to 44.7%, *p* < 0.001), and sleep disturbances showed a comparable pattern, affecting 54.8% of female vs. 45.3% of male PCS participants (*p* < 0.001, Table 2). In linear regression analyses modeling fatigue severity as a continuous outcome, female gender was associated with higher fatigue severity scores (*β* = 0.69, SE = 0.15, *p* < 0.001). This association remained statistically significant after adjustment for age, hospitalization status, vaccination status, and time since infection (*β* = 0.74, SE = 0.17, *p* < 0.001; Supplementary Table 1).

**Table 2:** Reporting of increased fatigue-related symptoms in PCS patient’s by gender.

Symptoms	Female *N* = 2,054	Male *N* = 490	Non binary *N* = 5	*p*-value
	Reported [*n*, (%)]	Reported [*n*, (%)]	Reported [*n*, (%)]	Reported [*n*, (%)]
Fatigue	1,099 (53.5)	227 (46.3)	1 (20.0)	0.005
Brain fog	1,128 (54.9)	219 (44.7)	3 (60.0)	<0.001
Sleep disturbances	1,125 (54.8)	222 (45.3)	1 (20.0)	<0.001

Symptoms were assessed on a Likert-scale (0–10), and a symptom was regarded as present if the severity was higher or equal to the median of the cohort. *p*-values derived by Fisher exact Test between female and male persons with PCS, corrected with Bonferroni-Holm method.

**Figure 2:**
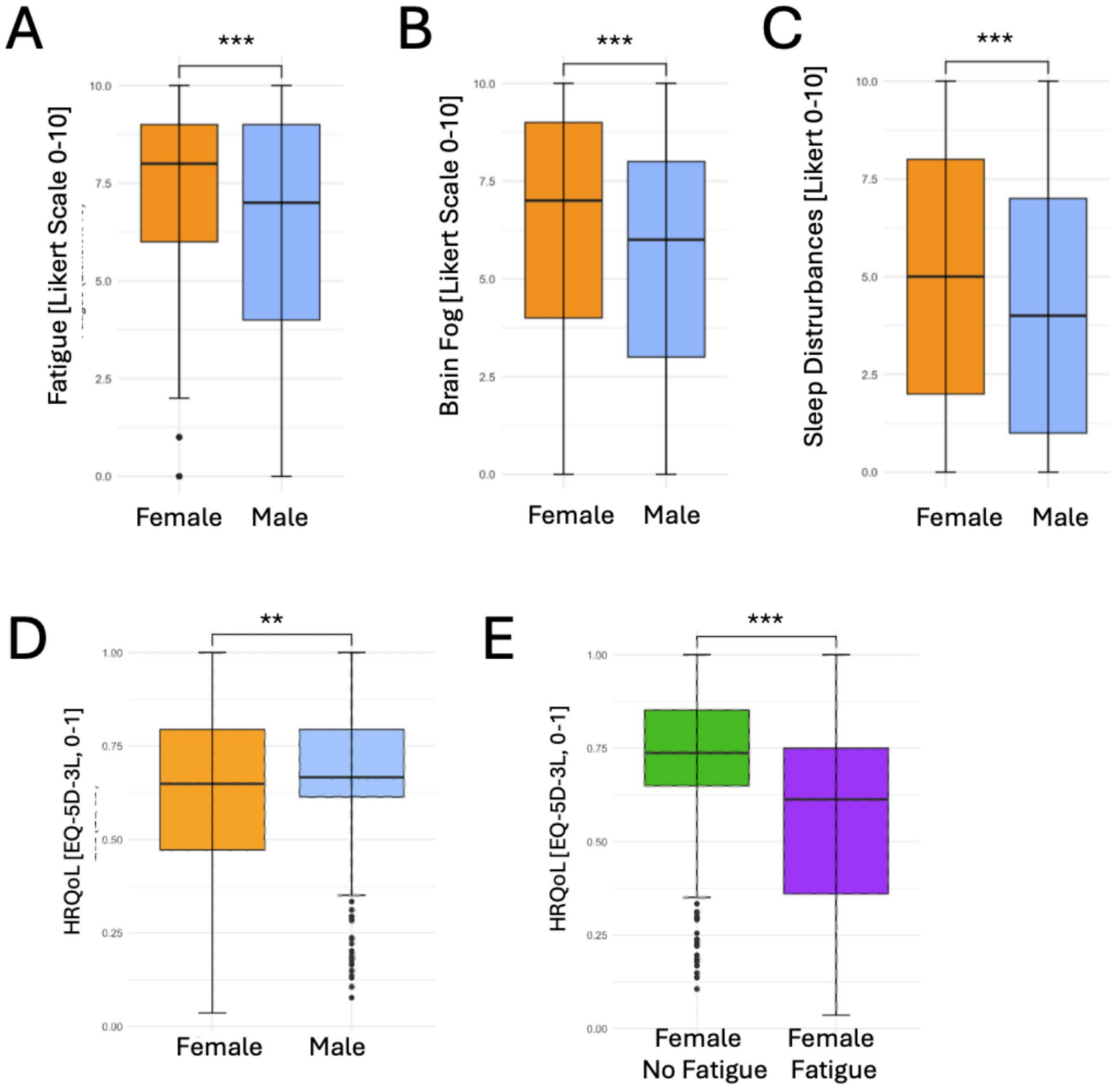
Fatigue and fatigue related symptoms and health related quality of life (QoL) in female vs. male respondents with PCS. **(A)** Fatigue, **(B)** Brain fog, **(C)** Sleep disturbances, **(D)** QoL per gender in perticipants with PCS, **(E)** QoL in female respondents with PCS with (green bar) and without (purple bar) fatigue scores above the median of the entire sample. Graphs display median +/− Q25-Q75, **p < 0.01, ***p < 0.001.

When examining the severity of these symptoms, female persons with PCS reported significantly higher fatigue intensity scores than their male counterparts [median 8 (IQR 6–9) vs. 7 (IQR 4–9), *p* < 0.001], and also brain fog and sleep disturbances were scored significantly higher by female persons with PCS [Figure 2; median of 7 (IQR 4–9) vs. 6 (IQR 3–8), *p* < 0.001 and 5 (IQR 2–8) vs. 4 (IQR 1–7), *p* < 0.001, respectively].

### Gender-specific health-related quality of life

Also, health-related quality of life was significantly lower in female participants with PCS compared to males (Figure 2D). The median EQ-5D-3L score was 0.65 (IQR 0.50–0.79) among females vs. 0.71 (IQR 0.61–0.90) in all males (*p* < 0.001). Non-binary participants had a median score of 0.66 (IQR 0.45–0.67). Importantly, female PCS participants with fatigue reported particularly low median levels of HRQoL which were significantly lower than those with PCS but without fatigue [0.56 (IQR 0.36–0.75) vs. 0.75 (IQR 0.65–0.90), *p* < 0.001, Figure 2E]. Also in male participants, those with fatigue reported lower median scores than those without fatigue [0.58 (IQR 0.39–0.74) vs. 0.79 (IQR 0.69–1.0), *p* < 0.001].

### Menstruation, PCS and fatigue in female participants

To further evaluate factors relevant in gender-dependent fatigue in PCS, we conducted a follow-up survey, which was answered by *n* = 260 female participants (median age 47 IQR 38–56), *n* = 45 male participants (median age 51 IQR42.5–59.5), and *n* = 3 non-binary persons (median age 29 IQR 22–36) with PCS. Again, higher fatigue severity scores were observed in female vs. male patients [median 8 (IQR 6–9) in female vs. 7 (IQR 4.5–8.5) in male patients].

**Figure 3:**
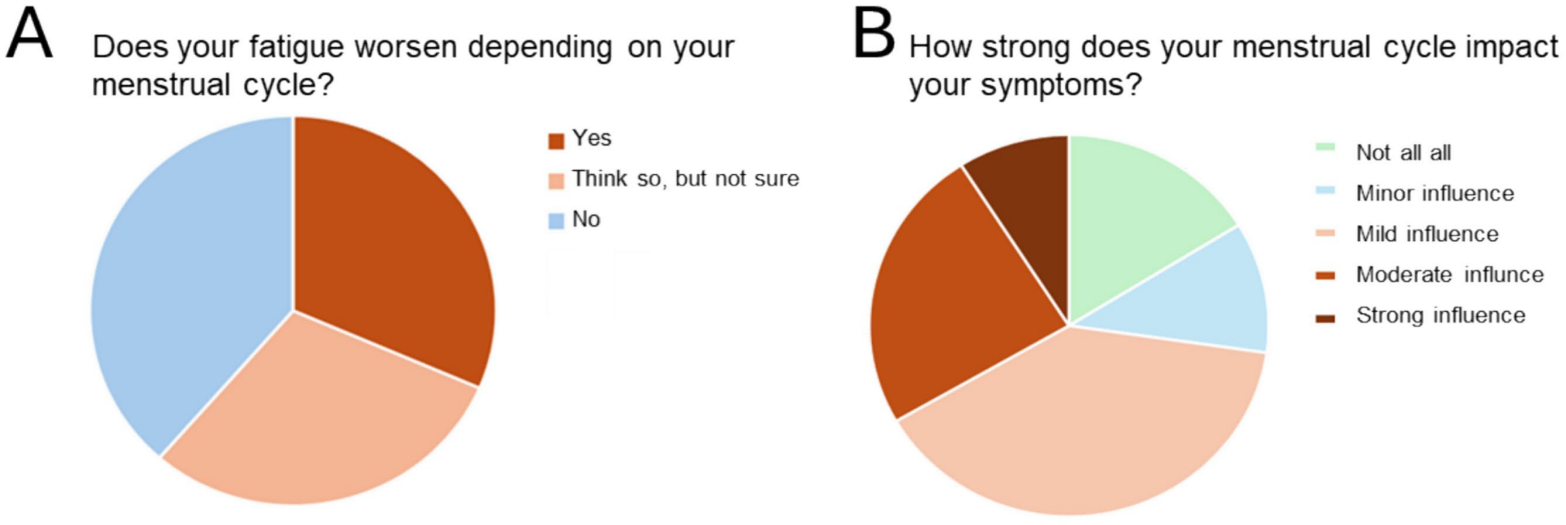
Menstrual cycle impacts fatigue symptoms in females with PCS. **(A)** Impact of menstrual cycle on fatigue in menstruating PCS patients. **(B)** Perceived strength of the menstrual cycles influence on PCS related fatigue.

When we asked whether the menstrual cycle impacted fatigue symptoms in menstruating persons (*n* = 131 responses), almost two thirds of respondents reported that they were either convinced (38.1%) or suspected (23.7%) that their menstrual cycle was associated with cyclical worsening of their fatigue symptoms (Figure 3A). Only 37% reported no impact of their menstrual cycle on fatigue symptoms. The impact of the menstrual cycle was rated to be mild in 11% of cases, but 39.3% of menstruating persons reported a moderate impact, and 33.3% reported a strong or very strong impact of their menstrual cycle on fatigue symptoms (Figure 3B).

## Discussion

This study provides findings of significant gender-based differences in fatigue-related symptoms among individuals with PCS, with females experiencing both higher prevalence and greater severity of these debilitating symptoms. The majority of menstruating persons with PCS in our survey reported moderate or strong association of their menstrual cycle on fatigue symptoms. Although our exploratory study cannot establish causality between gender and PCS-related symptoms, these findings have important implications for clinical care, research priorities, and public health approaches to PCS management.

The observed female predominance in fatigue-related symptoms aligns with broader patterns seen in other fatigue-related conditions such as chronic fatigue syndrome and fibromyalgia (20, 21). The significantly higher rates of fatigue, brain fog, and sleep disturbances in women with PCS suggest potential gender-specific mechanisms underlying these symptoms.

Our findings on self-reported menstrual cycle dependent symptom changes in PCS align well with previous research on the influence of female reproductive health on PCS symptoms (22, 23). A recent paper by Maybin et al. demonstrated that PCS symptoms fluctuate across the menstrual cycle, with severity peaking during perimenstrual and proliferative phases (24). Their research indicates this relationship stems from peripheral and endometrial inflammation. Our observation of higher fatigue severity in women with PCS and cyclical worsening of fatigue symptoms corroborates their findings suggests that hormonal fluctuations and associated inflammatory processes amplify fatigue and worsen HRQoL in affected women.

Although not assessed in our study, our results hint to several biological factors, that may contribute to these differences. Hormonal influences throughout the menstrual cycle, particularly estrogen and progesterone fluctuations, can significantly impact energy levels, sleep patterns, and cognitive function (25, 26). The hypothalamic–pituitary–adrenal axis, which plays a crucial role in stress response and energy regulation, shows sex and gender-specific differences that may influence fatigue and susceptibility to fatigue related syndromes (27, 28).

Neuroinflammatory processes and autoimmunity, increasingly recognized as central to PCS pathophysiology, also exhibit sex/gender-specific patterns (4, 29, 30). This may be due to differential immune cell activation and be associated with distinct risk profiles for postviral sequalae and neurological and neurodegenerative diseases in women vs. men (4, 31–33).

Our findings suggest several important clinical implications. In our cohort, HRQoL scores were low, particularly among participants experiencing fatigue and among women. These scores were below the reference values reported for the older German population but comparable to index values observed in patients with rheumatoid arthritis (34, 35). Sex/gender differences in HRQoL have been described for different diseases (36), but it remains unclear whether this is primarily due to higher disease burden or other factors. The substantially poorer HRQoL scores among female survey participants with PCS in our cohort - surpassing a minimally important difference when compared to male respondents - highlight the urgent need for sex and gender-sensitive clinical approaches (37). Healthcare providers should be aware of the higher likelihood and severity of fatigue symptoms in female PCS patients and adapt management strategies to possible symptom worsening during specific menstrual phases. To our current knowledge, such interventions might include specialized fatigue management programs, cognitive behavioral therapy adapted for PCS-related cognitive symptoms, or hormonal evaluations in selected cases (38–41).

Our findings underscore the ongoing need for research to systematically examine sex/gender as a central biological variable in PCS, as we focused on the self-reported gender. Mechanistic analyses investigating biological aspects such as hormonal influences, particularly longitudinal relationships between hormonal factors and symptom severity, will provide further insight into PCS pathophysiology and may disclose novel treatment targets.

Several limitations of our work should be acknowledged. Although women are more likely to experience PCS (2), the predominantly female composition of our cohort may reflect selection bias, which limits the generalizability of our findings. Women may be more likely to seek care and take differential approaches to participate in health research (42, 43), and the dominance of female participant might lead to an overestimation of gender-specific differences. Also, we asked participants about their self-perceived gender and not biological sex, which is non-aligning in an estimated 0.4–1.6% of individuals (16, 17). The analysis of gender identity represents a methodological simplification, especially when describing the non-binary group, and should be taken into account when interpreting our results in the context of work that was performed on sex and not gender stratified cohorts. Also, the influence of important factors known to differ significantly between genders such as socioeconomic status, caregiving responsibilities, and mental health (44) were not or only in part analyzed in and should be assessed in future studies focusing on gender specific disease burden in PCS. The self-reported nature of symptom data introduces potential reporting bias, though validated questionnaires were used to minimize this concern. Fatigue severity was dichotomized at the median for the primary analyses due to the absence of validated clinical thresholds for post-COVID symptom severity. This approach may influence prevalence estimates and attenuate or exaggerate effect sizes. To address this, sensitivity analyses using continuous symptom scores were performed and yielded consistent results. The cross-sectional design prevents causal inferences about gender-related mechanisms underlying fatigue differences. Also, we did not ask for further factors crucially impacting reproductive and female health such as for example endometriosis, which was recently shown to significantly influence PCS symptoms as well (45). Our cohort was well educated, with 61.8% with a college-preparatory qualification, which makes the generalization to persons with lower education or socioeconomic status challenging.

Our findings underscore the critical need for research to systematically examine gender as a central biological variable in PCS. Future investigations should prioritize longitudinal studies examining hormonal fluctuations across menstrual cycles and their relationship to symptom severity. Also, the influence of factors such as hormonal contraception, detailed symptoms of (peri-) menopause, or conditions such as endometriosis should be studied, as well as biomarkers that account for sex-specific inflammatory and neuroendocrine responses. Despite its limitations, our study provides compelling evidence that gender-based differences in PCS-related fatigue are not merely quantitative but reflect fundamental biological variations that demand distinct clinical approaches. The substantial symptom burden experienced by females, compounded by menstrual cycle-dependent fluctuations affecting two-thirds of menstruating persons with PCS, necessitates a paradigm shift from one-size-fits-all protocols to precision medicine approaches that integrate reproductive health into PCS management. Only through recognizing and addressing these gender-specific patterns can we move beyond symptomatic treatment toward truly personalized therapeutic strategies that acknowledge the unique biological realities of women with PCS, ultimately transforming post COVID care from a universal model that inadvertently disadvantages women to one that provides equitable, effective treatment tailored to each patient’s biological and hormonal context.

## Data Availability

The raw data supporting the conclusions of this article is a topic of current research, but will be made available by the authors upon reasonable request.
