# Supplementary material for: Post-COVID fatigue disproportionately affects women: evidence from the DEFEAT Corona cohort
Source: Front Public Health. 2026 Feb 16;14:1755106. doi: 10.3389/fpubh.2026.1755106 (PMC12950731; doi:10.3389/fpubh.2026.1755106)
Supplement: Supplementary file 1 [file Table_1.docx]

**Suppl. Table 1: multilinear regression model for fatigue symptoms**

|  | Estimate (SE) | P-value | CI |
| --- | --- | --- | --- |
| Intercept | 4.09 (0.46) | <0.001 | 3.20 – 4.99 |
| Gender (Female) | 0.741 (0.17) | <0.001 | 0.41 – 1.08 |
| Age | 0.008 (0.01) | 0.158 | −0.00 – 0.02 |
| Vaccinated before infection | 0.779 (0.212) | <0.001 | 0.36 – 1.20 |
| Hospitalization | 0.829 (0.28) | 0.003 | 0.28 – 1.38 |
| Time since infection in weeks | 0.018 (0.003) | <0.001 | 0.01 – 0.03 |

*SE = standard error, CI = Confidence interval, P-values<0.05 were considered significant.*
